# Baculovirus Variant Detection from Transient CRISPR-Cas9-Mediated Disruption of *gp64* at Different Gene Locations

**DOI:** 10.3390/ijms26125805

**Published:** 2025-06-17

**Authors:** Madhuja Chakraborty, Lisa Nielsen, Delaney Nash, Mark R. Bruder, Jozef I. Nissimov, Trevor C. Charles, Marc G. Aucoin

**Affiliations:** 1 Department of Chemical Engineering, University of Waterloo, 200 University Avenue West, Waterloo, ON N2L 3G1, Canada; m6chakraborty@uwaterloo.ca (M.C.); lisa.beth.coles@uwaterloo.ca (L.N.);; 2 Department of Biology, University of Waterloo, Waterloo, ON N2L 3G1, Canada; d2nash@uwaterloo.ca (D.N.); jnissimov@uwaterloo.ca (J.I.N.); trevor.charles@uwaterloo.ca (T.C.C.)

**Keywords:** baculovirus, CRISPR-Cas9, tiled-amplicon sequencing, bioinformatics pipeline, variant calling pipeline, transfection-infection assay (T-I assay), whole genome sequencing, next-generation sequencing, p6.9 promoter, AcMNPV

## Abstract

The Baculovirus Expression Vector System (BEVS) is an important protein and complex biologics production platform. The baculovirus GP64 protein is the major envelope glycoprotein that aids in virus entry and is required for cell-to-cell transmission in cell culture. Several studies have developed strategies around *gp64* gene disruption in an attempt to minimize baculovirus co-production. Here, we investigate the result of transiently targeting the baculovirus *gp64* gene with CRISPR-Cas9 during infection. Because not all genomes are effectively disrupted, we describe a variant calling methodology that allows the detection of the targeted mutations in *gp64* even though these mutations are not the dominant sequences. Using a transfection-infection assay (T-I assay), the AcMNPV *gp64* gene was targeted at six different locations to evaluate the effects of single and multiple targeting sites, and we demonstrated a reduction in the levels of baculovirus vectors while maintaining or enhancing foreign protein production when protein was driven by a p6.9 promoter. Viral genomes were subsequently isolated from the supernatant and cell pellet fractions, and our sequencing pipeline successfully detected indel mutations within *gp64* for most of the single-guide RNA (sgRNA) targets. We also observed that 68.8% of variants found in the virus stock were conserved upon virus propagation in cell culture, thus indicating that they are not detrimental to viral fitness. This work provides a comprehensive assessment of CRISPR-Cas9 genome editing of baculovirus vectors, with potential applications in enhancing the efficiency of the BEVS.

## 1. Introduction

The baculovirus expression vector system (BEVS), consisting of an insect cell line and recombinant baculovirus expression vectors (rBEVs), was first utilized for the production of biologically active human interferon β [1] in the 1980s. This study paved the path for numerous applications of the BEVS, including the production of recombinant proteins, commercial vaccines, virus-like particles (VLPs), adeno-associated virus-vectors, and even its use as a BacMam viral vector for gene therapy [2,3,4,5,6,7]. The ability of insect cells to perform mammalian-like post-translational modifications (PTMs), the inherent biosafety of baculoviruses, the ability of baculoviruses to accept large DNA insertions, and the resulting overexpression of genes inserted in the *polyhedrin* locus of the rBEV, make the BEVS an attractive platform for laboratory research and industrial production [6,8]. Among baculoviruses, *Autographa californica* multiple nucleopolyhedrovirus (AcMNPV) is the most widely used baculovirus in the BEVS [9]. The AcMNPV C6 strain was the first baculovirus to be fully sequenced [10]. It has a double-stranded DNA (dsDNA) genome of approximately 134 kbp in size and 155 open reading frames (ORFs) [10,11]. Despite the widespread application and numerous advantages of the BEVS, the co-production of recombinant baculoviruses in insect cell cultures complicates the separation and purification of many desired products.

The budded virus (BV) phenotype is responsible for baculovirus propagation in cell cultures [12] and the GP64 glycoprotein, found in Group I nucleopolyhedroviruses (NPVs) such as the AcMNPV, is used as the membrane fusion protein for virus entry [9,13]. This low pH-activated protein is one of the most abundant proteins in the AcMNPV BV envelope, and its deletion impairs the budding of progeny viruses from cells [12,14,15,16,17]. Strategies for eliminating GP64 using a transcomplementing cell line strategy [18] has not seen widespread use, likely due to problems in generating high titer stocks needed for the infection process. Recently, CRISPR-Cas9 disruption of *gp64* has been used to reduce progeny virus production in cell cultures, improving the quality of the resulting downstream processing feed [19,20,21,22].

More recently, we reported on the consensus sequence of an rBEV that contains a green fluorescent protein (GFP) gene that is used in CRISPR-Cas9 gene-targeting studies [21]. However, genomes transiently targeted during an infection do not result in changes to a majority of the genomes. This may be in part due to an imbalance in the kinetics of baculovirus replication and single-guide RNA (sgRNA)-Cas9 targeting-cleaving, or may be more directly related to reduced *cas9* expression post-infection because the expression is driven by an OpIE2 promoter [23]. Transiently targeted baculovirus genomes are therefore more likely to behave like virus variants in human populations. Here, we aim to demonstrate (through sequencing) that the phenotypic changes observed are due to CRISPR-Cas9-mediated targeted disruption and that the *gp64* gene can be targeted at multiple locations. We also describe a variant calling bioinformatics pipeline that successfully confirms mutations within the targeted *gp64* gene. Additionally, while investigating the variants, we observed that there are certain regions, such as the homologous repeat regions (hrs), where mutations are consistently present; that is, they are carried over as variants between viral passages.

## 2. Results

### 2.1. Effect of gp64 Gene Disruption on Foreign Protein and Progeny Virus Production

**Figure 1:**
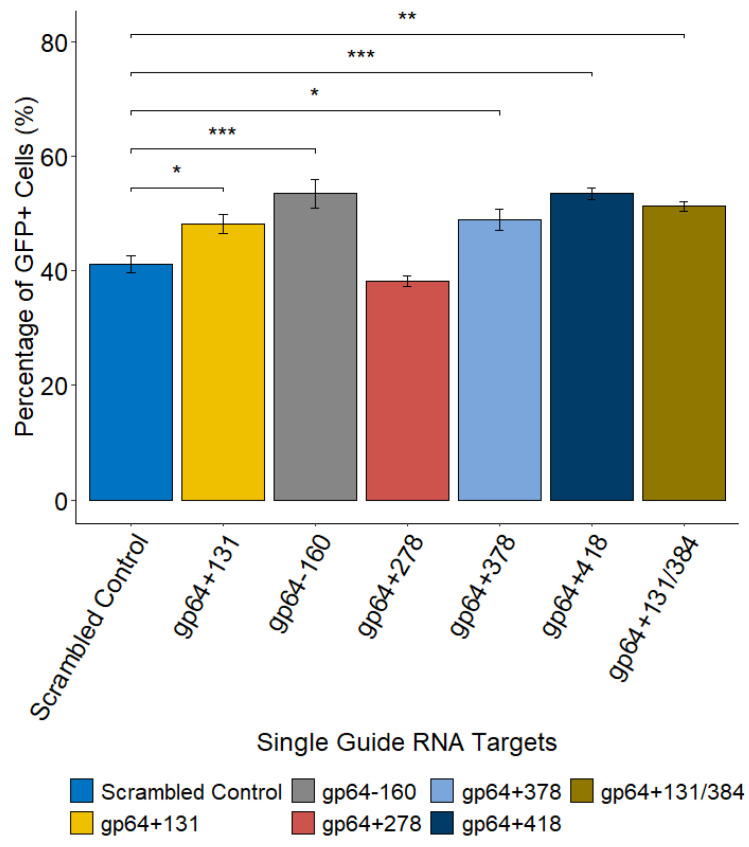
Effect of CRISPR-Cas9-mediated gene disruption of the AcMNPV *gp64* gene at single or dual locations on GFP production. The high fluorescence bin data from 3 biological replicates for each target and the scrambled control are shown, where the error bars represent the mean ± standard error (within groups). ANOVA with Dunnett’s test was performed to compare each target to the scrambled control. ***, *p*-value < 0.001; **, *p*-value < 0.01; and *, *p*-value < 0.05.

**Figure 2:**
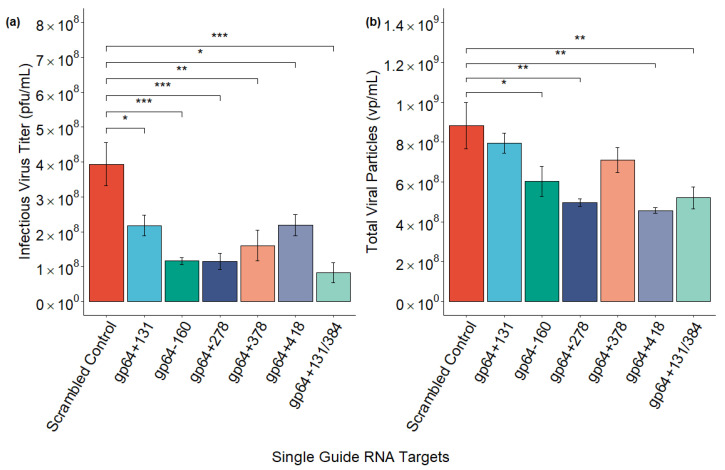
Impact of CRISPR-Cas9 targeting of the AcMNPV *gp64* gene using single or dual sgRNA targets on (**a**) infectious virus titer (IVT) and (**b**) total viral particles. Data from 3 biological replicates for the scrambled control and each target are presented with error bars for the mean ± standard error (within groups). ANOVA with Dunnett’s test compared each target to the scrambled control. ***, *p*-value < 0.001; **, *p*-value < 0.01; and *, *p*-value < 0.05.

The CRISPR-Cas9-based transfection-infection assay (T-I assay) [20] was used to disrupt the AcMNPV *gp64* gene at different locations using single or dual sgRNA targets, with one or two *gp64* targeting spacer sequences, respectively. Disruption of the *gp64* gene resulted in similar fluorescence for all six targets compared to the scrambled control. While the percentage of cells expressing GFP was enhanced for most of the targets (gp64+131, gp64−160, gp64+378, gp64+418, and gp64+131/384), it was slightly lower (not significant) for the gp64+278 target as compared to the control (Figure 1). On the other hand, disrupting *gp64* reduced the infectious virus titer (IVT) for all the targets as expected, with the maximum decrease observed for the dual sgRNA target (Figure 2a). Additionally, the total viral particles, including infectious and non-infectious virions, dropped upon *gp64* disruption compared to the scrambled control (Figure 2b).

### 2.2. Confirmation of CRISPR-Cas9-Mediated Gene Editing of gp64

CRISPR-Cas9 targeted rBEV genomes were analyzed to confirm the cut sites and mutations within the *gp64* gene. A shotgun-sequenced rBEV genome [21] served as the reference genome for data analysis. Table 1 gives an overview of the estimated and observed indel positions in *gp64* as well as the observed *gp64* mutations and variant frequencies. While observed mutation positions fell within the expected range for targets gp64+131, gp64−160, gp64+378, and gp64+418, no mutations were detected within the target region for gp64+278 and the dual gp64+131/384 (Table 1). Interestingly, for target gp64−160, true mutations (Fisher’s exact test *p*-value ≤ 0.05) were identified at multiple sites in the estimated range, which could potentially be variants of this gene disruption.

Specifically, targeted disruption of *gp64* resulted in deletion mutations within the *gp64* gene for most of the sgRNA targets (Table 1). Importantly, no mutations were observed in the *gp64* gene for both scrambled and infected-only controls. A close look at the deletion mutations within *gp64* revealed that the same mutations occurred in the intracellular (cell pellet) and extracellular (supernatant) fractions for each target except gp64+131 and gp64−160 (Table 1). The gp64−160 target led to three additional deletions within *gp64* when the gDNA was extracted from the supernatant compared to the cell pellet, thus resulting in a total of five possible variants. For the gp64+131 target, a deletion appeared in the cell pellet fraction, but no mutation was detected in the supernatant fraction by our sequencing pipeline. Table 1 also shows the frequencies at which each of these observed mutations occurred in the cell pellet and supernatant fractions of the *gp64* targeted genomes. While the lowest detected frequency was 1.43%, the maximum frequency was 8.47%, both corresponding to variants obtained from targeting *gp64* with the gp64−160 target.

### 2.3. Variant Generation or Conservation over Passages

To investigate whether variants are a product of virus amplification in cell culture or are conserved over passages, we ran the variant calling pipeline for the p6.9GFP rBEV amplified in Sf9 cells in suspension, utilizing the consensus sequence of the same rBEV (shotgun-sequenced rBEV) [21] as the reference genome. Compared to the reference genome, 141 true mutations were detected when the rBEV was analyzed for variants (hereby referred to as virus stock variants). It is to be noted that upon variant analysis of the rBEV, most of the mutations were in the three hrs, hr1, hr2, and hr3, with no mutations detected within the *gp64* gene. While 44 mutations were unique to the virus stock, 97 mutations were carried over during the T-I assay, as observed in the genomes recovered from the T-I assay, which were sequenced by the tiled-amplicon sequencing assay and analyzed by the variant calling pipeline. Specifically, the majority of the conserved mutations in the T-I assay variants were in the different hrs (hr1, hr2, and hr3), with only 14 of them residing outside these repetitive regions (Table 2). The common variants observed in both the shotgun sequenced virus stock and the tiled-amplicon-sequenced gDNAs from the T-I assay can be considered variants that are preserved upon virus propagation in Sf9-Cas9 cells, and they may not be detrimental to virus fitness, as they were observed again as variants.

### 2.4. Evaluation of Mutations Outside the Targeted gp64 Gene

**Figure 3:**
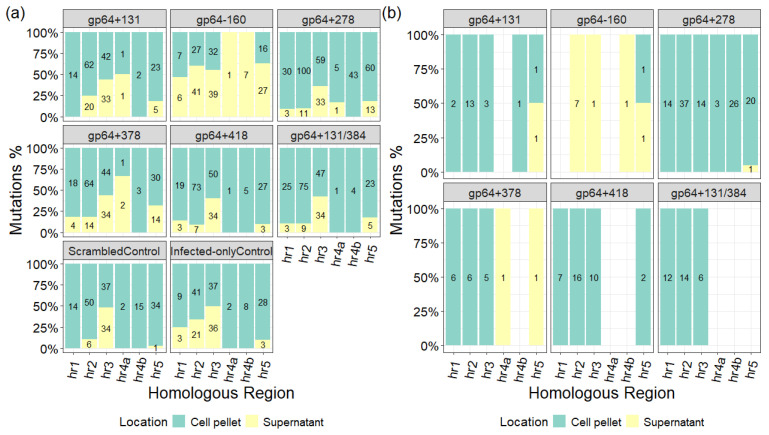
(**a**) Total and (**b**) filtered (excluding controls and conserved variants) mutation distribution in the different hrs of gDNA pools are highlighted here. The numbers within each bar represent the number of mutations observed in hrs for gDNA pools extracted from the cell pellet or supernatant of a sample. Each sample was run in duplicate and sequenced at a depth of 400×. Only true mutations with Fisher’s exact test *p*-value ≤ 0.05 are considered.

Mutations in the viral genomes were also detected outside the targeted *gp64* gene following the CRISPR-Cas9-based T-I assay. However, as we are assessing variants in a heterogeneous pool of gDNAs, these untargeted mutations do not necessarily exist in the same genome where the *gp64* mutations were observed. A majority of these mutations were confined to the hrs; specifically, in six hrs (Figure 3a) out of the nine hrs in the AcMNPV C6 genome [10]. The hrs with no mutations (hr1a, hr2a, and hr4c) are 18–30 bp long, while the ones with mutations are at least 150 bp long. Moreover, except for the gp64−160 target sample, the majority of the hrs mutations were either found exclusively in the gDNA pools extracted from the cell pellets, or distributed almost equally between the cell pellet and supernatant fractions (Figure 3a). It was further observed that most hrs mutations in the targeted samples were either common with the controls or carried over from the virus stock, as depicted by the drop in mutation counts between Figure 3a,b. Additionally, filtering out these common or preserved mutations revealed that not all targeted samples exhibited unique mutations in all six hrs, and except for the gp64−160 target sample, most of the mutations were in the cell pellet fractions (Figure 3b).

**Figure 4:**
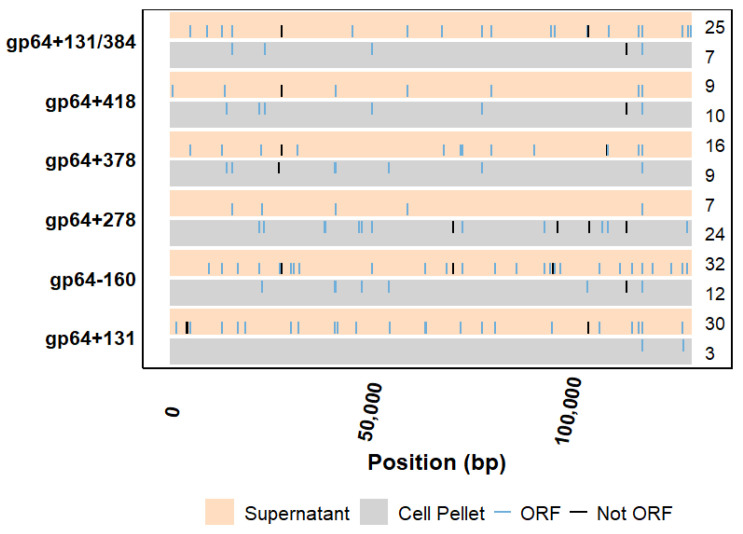
Overview of mutations in the pool of gDNAs outside the hrs and the CRISPR-Cas9 targeted *gp64* gene. The numbers on the right side of each horizontal bar represent the total number of true mutations (Fisher’s exact test *p*-value ≤ 0.05) observed after excluding controls and preserved variants. The blue and black vertical lines are the corresponding mutation positions within and outside ORFs, respectively, unique to the targeted samples. Each sample was run in duplicate and sequenced at a depth of 400×.

Mutations outside the hrs and the targeted *gp64* gene were observed in both the sgRNA target samples and the controls. However, these mutations exist within viruses that constitute a small fraction of the total number of viruses (Figure 4). CHOPCHOP’s uniqueness method, which looks for mismatches in 20 bp sequences upstream of protospacer adjacent motifs (PAMs) [24,25], returned that there were no predictable off-targets for the sgRNA targets used in this study. It was further observed that for the sgRNAs to bind to an off-target site, at least four mismatches for gp64+131, five mismatches for gp64−160, five mismatches for gp64+278, four mismatches for gp64+378, five mismatches for gp64+418, and six mismatches for gp64+131/384 were required. However, they lacked the PAM sequence, without which CRISPR-Cas9-mediated binding and cleavage would not occur.

## 3. Discussion

Over the last two decades, the BEVS platform has been utilized for the production of ten human and five veterinary commercially approved biologics, with many other biologics currently in different phases of clinical trials [26,27]. However, a drawback of the platform is the co-production of virions and the products of interest, like enveloped VLPs or recombinant proteins. Deletion or inactivation of genes essential for budded virus release in the final production stage can reduce baculovirus contamination [19]. The AcMNPV *gp64* gene codes for the major envelope fusion protein, GP64, which is essential for cell-to-cell transmission of the virus and systemic infection of the host [9,17]. A previous study reported that CRISPR-Cas9-mediated disruption of *gp64* reduced IVT by 99% while maintaining Gag VLP production, thus demonstrating the use of CRISPR-Cas9 to address BV co-production in cell culture supernatant [19]. While previous works on CRISPR-Cas9-mediated *gp64* disruption analyzed only the phenotypic changes [19,20,22], we investigated both phenotypic and genotypic changes with a focus on the genomic changes that occurred upon CRISPR-Cas9 genome editing using *gp64* as an example. In this study, we used a CRISPR-Cas9-based T-I assay (screening assay) [20] to disrupt the *gp64* gene at different locations (five single sgRNA targets and one dual sgRNA target) and evaluated its impact on GFP and progeny virus production. The percentage of GFP-expressing cells (Figure 1) upon *gp64* gene disruption was similar to the control for all sgRNA targets while reducing the IVT and total viral particles (Figure 2), which is consistent with previous reports [19,20,21]. Our data also suggests that a synchronous infection at a multiplicity of infection (MOI) of 3 pfu/cell does not reduce foreign protein production while transiently targeting *gp64*. This demonstrates the need for a different approach to low MOI strategies, one that may require using a subset of non-Cas9 expressing cells at the beginning of production. No matter what target site was chosen, similar outcomes were observed. Thus, the CRISPR-Cas9-mediated targeting of *gp64* is a promising strategy to reduce rBEV contamination without affecting foreign protein production when using the p6.9 promoter to drive foreign protein expression.

### 3.1. CRISPR-Cas9-Mediated Targeted Disruption of gp64 During Virus Propagation

The CRISPR-Cas9 system uses a short RNA to guide the Cas9 endonuclease to a specific genomic location, where Cas9 then cleaves double-stranded DNA. Once the DNA is cleaved, cellular repair mechanisms will try and repair the break, which can result in indel mutations. If the cell is supplied with complementary DNA, the repair can result in “designed/premeditated” changes; otherwise, the changes should be random. As a result, the CRISPR-Cas9 system can be used for targeted gene editing [28,29]. The indel mutations that occur are located 3–4 bp upstream of the PAM and can range between 1 and 5 bp and lead to targeted gene disruption [30,31,32]. Although we observed phenotypic effects (fluorescence and virus titer), we wanted to confirm that our observations were due to the disruption of our intended target. A secondary goal was to investigate if the mutations were confined to unpackaged (intracellular) or budded (extracellular) baculovirus genomes, or if they could be observed in both fractions.

It was postulated that our targeted rBEV genomes might have constituted a minority population. Thus, to analyze mutations upon gene disruption, we used a variant calling pipeline based on the computational tool iVar (intrahost variant analysis of replicates), originally used for measuring intrahost Zika and West Nile virus diversity [33]. In this study, different thresholds were used for the variant calling pipeline parameters to ensure the detection of high-quality, low-frequency variants. For instance, the *samtools view* –q and –F parameters filtered out alignments with a low mapping quality score below 10 and excluded unmapped reads, as well as secondary (suboptimal) and supplementary (segmented) alignments from downstream analysis using the 2308 flag [34]. Additionally, *samtools ampliconclip* ensured that primers were trimmed from both ends of mapped reads, while the –filter-len parameter excluded short reads below 200, which are often less reliable [34]. In the *iVar variants* step, the –q parameter was used to set the minimum base quality score to 20 (default) for a position to be considered in variant calling, and the –t parameter defined the variant calling frequency threshold, enabling the detection of low-frequency variants (present in at least 1%) [33].

A drawback of the plasmid-based delivery of sgRNA is that transfection is not 100% efficient, which can result in some cells not receiving the sgRNA. Additionally, the AcMNPV *gp64* gene was targeted while the virus was undergoing its infection cycle in Sf9-Cas9 cells, where the viral genome will be undergoing replication. Thus, it is possible that the double-stranded breaks (DSBs) can undergo homology-directed repair (HDR) using other copies of the genome as a DNA template. Using the variant calling pipeline with specified thresholds, we were able to identify mutations within the expected regions of the *gp64* gene for most of the sgRNA targets, and these mutations were observed in the cell pellet as well as the supernatant fractions (Table 1). However, even though expected phenotypic changes were observed, no *gp64* mutations were detected for the dual sgRNA target gp64+131/384 and the single sgRNA target gp64+278, as well as for the supernatant fraction of the single sgRNA target gp64+131. It is possible that targeting the *gp64* gene at two locations within close proximity induced DSBs that were beyond repair by non-homologous end joining (NHEJ) or HDR. Consequently, while the intracellular and extracellular viral genomes from the rBEV infecting untransfected Sf9-Cas9 cells (no sgRNA) were sequenced, the targeted genomes were probably routed for destruction by nucleases. Moreover, for the gp64+278 target and the supernatant fraction of the gp64+131 target, perhaps only properly repaired or untargeted genomes were detected, and the targeted genomes with indels were present in an even lower frequency (less than 1%). Applying less stringent thresholds, such as lowering *samtools ampliconclip* –filter-len to 50 and *iVar variants* –q to 10 or 15, did not reveal mutations within *gp64* for the targets where mutations were not initially detected.

### 3.2. Are CRISPR-Cas9 Off-Targets Observed in Our System?

The CRISPR-Cas9 genome-editing technology is often associated with off-target effects, where the Cas9 endonuclease cleaves unwanted genetic locations that are similar to the target and have the required PAM. In this study, sgRNAs were designed using the online tool CHOPCHOP with advanced parameters, and only those sgRNAs that returned no off-targets were selected [24,25,35]. A previous study demonstrated that while Cas9 tolerated 2–3 interspaced or concatenated mismatches in the PAM distal region, even 2 mismatches, whether interspaced or concatenated, in the PAM seed region considerably reduced or abolished Cas9 activity [25]. For Cas9 to cleave an off-target site, the appropriate PAM must be present [25]. To further investigate whether our sgRNA targets would have off-target effects, we scanned our reference genome (on Benchling [Biology Software] (2025), retrieved from https://benchling.com) for each sgRNA target by increasing the number of mismatches between the 20 bp spacer sequence and the reference genome. We noticed that a minimum of 4 mismatches were required for our selected sgRNA targets to guide the Cas9 to unintended genomic locations. Further probing these locations revealed missing PAM sites, thus leading to no Cas9 binding and cleavage.

### 3.3. Variant Conservation or Random Mutations upon Virus Propagation in Cell Culture

As we are analyzing variants in this study, we wanted to investigate if any of the variants from the T-I assay were present in our p6.9GFP rBEV stock. Two different sequencing methodologies, shotgun and tiled-amplicon sequencing, were used for the virus stock and rBEV genomes recovered from the T-I assay, respectively. Applying the variant calling pipeline to both sequencing reads revealed that 68.8% of the virus stock variants were carried over to the T-I assay. While these variants were mainly confined to the hrs (hr1, hr2, and hr3), ~12.6% of them were present in other regions of the genome. These mutations do not form the consensus sequence of the virus stock or the genomes from the T-I assay but are rather present as variants. Additionally, since they are conserved as variants, it suggests that they do not negatively impact virus fitness.

Baculovirus genomes are susceptible to mutations originating from replication errors during propagation in insect cell cultures [11,36]. These random mutations are often observed in the hrs as well as other regions of the genome [37]. The AT-rich hrs are repetitive in nature and considered mutation hotspots due to their susceptibility to error-prone processes during viral replication [37,38,39]. Our data suggested that the longer hrs (≥150 bp) were more prone to mutations compared to the shorter ones (18–30 bp). It is important to note that the bioinformatics pipeline used in this study was designed to identify variants (minor species) within a heterogeneous population; thus, these mutations do not represent the consensus sequences of the viral gDNAs and are not necessarily present in the same genome where *gp64* mutations exist. Variants detected outside the targeted *gp64* gene could result from baculoviruses’ susceptibility to mutations during amplification in insect cell cultures [11,37]. Moreover, most of the variants (outside *gp64*) in the targeted samples were also observed in the controls or were conserved over passages.

A previous study demonstrated that continuous propagation of an AcMNPV E2 strain-based recombinant baculovirus in Sf21 cells, at a high MOI of 20 pfu/cell, led to a drop in titer with complete loss observed from P31; and this was associated with the formation of defective interfering particles (DIPs) that can infect cells but are not capable of completing the infection cycle [37]. Although DIP generation in high-passage viral stocks has been reported upon serial passages at high MOI in insect cell cultures, the questions remain whether the viruses need to be amplified at high MOI during the seed train and whether such a high-passage stock is required for production in bioreactors. Typically, infecting at a low MOI of 0.1 pfu/cell until the production stage would suffice to obtain a virus stock for production. To put that into perspective, 5 mL of a P1 virus stock with a titer of 1 × 10^8^ pfu/mL can infect 2.5 L of culture seeded at a density of 2 × 10^6^ cells/mL at an MOI of 0.1 pfu/cell. Following that, 50 L culture seeded at 2 × 10^6^ cells/mL can be infected with 2.5 L of the P2 stock (~4 × 10^8^ pfu/mL) at an MOI of 10 pfu/cell to achieve synchronous infection. Thus, realistically, one can say that a high-passage virus stock is not necessary to generate enough stock to infect cells in a bioreactor. Since, in our work, we infected the Sf9-Cas9 cells with a low passage (P2) p6.9GFP rBEV stock at a moderate MOI of 3 pfu/cell, we believe that the mutations observed outside the targeted region were not due to the formation of DIPs. These deletion mutations outside *gp64* were, in fact, a maximum of 20 bp (within *egt*) or 19 bp (within hr2) long compared to large genomic deletions of ~43% in DIPs [37,40]. Additionally, it was previously demonstrated that the 20 bp deletion in *egt* originates from a small shotgun sequencing reference genome assembly error, where this 20 bp region should not be present in the reference genome [21].

## 4. Materials and Methods

### 4.1. Cell Line and Maintenance

Cas9 expressing Sf9 (Sf9-Cas9) cells [23] and parental Sf9 cells were maintained in suspension in Sf-900^TM^ III serum-free media (SFM) (Gibco, Carlsbad, CA, USA) at 27 °C and 130 rpm. To keep the cells in the exponential growth phase, they were passaged every 3–4 days until the viable cell density reached between 3 and 5 × 10^6^ cells/mL. Since the Sf9-Cas9 cells carried a Cas9-2A-puromycinR gene cassette, puromycin (Sigma-Aldrich, Oakville, ON, Canada) was routinely added to the Sf9-Cas9 cell cultures at a concentration of 5 µg/mL to ensure *cas9* expression.

### 4.2. Plasmid Design and Construction

All primers used in this work were synthesized by Integrated DNA Technologies (IDT) (Coralville, IA, USA) and listed elsewhere [35]. sgRNA plasmids were constructed using the NEBuilder 2× HiFi DNA assembly master mix (New England Biolabs, Whitby, ON, Canada) as previously described [35]. Briefly, to obtain the SfU6-sgRNA insert, a fusion PCR was performed with a PCR-amplified SfU6 promoter [41] gBlock (synthesized dsDNA) gene fragment (IDT) and a gRNA scaffold with a scrambled spacer sequence (Table 3) at the 5^′^ end and a transcriptional terminator at the 3^′^ end (Addgene # 49411) [42] as templates. Separately, the ampicillin resistance gene (ampR) and the pBR322 origin of replication (ori) from the pBR322-TIMER plasmid (Addgene # 103056) [43] were PCR-amplified to obtain the backbone fragment. The pSfU6-sgRNA scrambled control plasmid was constructed in a two-fragment Gibson assembly reaction utilizing the SfU6-sgRNA insert and the backbone fragment.

Re-targeting sgRNA plasmids were constructed using the pSfU6-sgRNA scrambled control plasmid as the template for inverse PCR [20]. Primers with an altered spacer sequence (for different locations within the *gp64* gene), appended to their 5^′^ ends and annealing either to the 5^′^ end of the gRNA scaffold or the 3^′^ end of the SfU6 promoter, were used to amplify the entire sgRNA plasmid as a linear fragment. Following DpnI digestion to remove the template DNA, the gel-extracted re-targeting sgRNA fragment was re-circularized. The new spacer sequence at both ends acted as the homologous regions for Gibson assembly. The online tool “CHOPCHOP” [24] was used to design AcMNPV *gp64* targeting spacer sequences utilizing the previously outlined spacer sequence selection criteria [35]. The gp64+131 sgRNA plasmid was obtained from a previous study [20]. All the spacer sequences along with their PAM sequences used in this study are listed in Table 3.

### 4.3. Baculovirus Amplification and Quantification

A previously constructed rBEV carrying the GFP monomeric Azami green under the AcMNPV p6.9 promoter (herein referred to as p6.9GFP rBEV) was used in this study [20]. A working virus stock was obtained by amplifying a P1 stock of this rBEV in Sf9 cells at a low MOI of ~0.1 pfu/cell until the viable cell density dropped between 80 and 90%.

Using an end-point dilution assay (EPDA), IVTs were quantified as previously described [44,45]. Briefly, 96-well tissue-culture-treated plates (VWR International, Mississauga, ON, Canada) were seeded with 100 µL of Sf9 cells diluted to 2 × 10^5^ cells/mL in Sf-900^TM^ III SFM. The cells were allowed to adhere for around 1 h at 27 °C. In the meantime, dilutions of the virus stocks were prepared by serial dilution from 10^−2^ to 10^−8^ in Sf-900^TM^ III SFM. Following the incubation period, cells were inoculated with 10 µL of a virus dilution, resulting in 12 replicates per dilution per row, with an uninfected row receiving no virus. Finally, the plates were observed under a fluorescence microscope after being incubated at 27 °C for 6–7 days to determine the fluorescence. The virus titer in plaque-forming units per mL (pfu/mL) was then obtained by taking the reciprocal of TCID_50_ expressed in mL of virus added and multiplying it by 0.68 based on the Poisson distribution.

### 4.4. Transfection-Infection Assay (T-I Assay)

The AcMNPV *gp64* gene was targeted via a previously developed CRISPR-Cas9-based T-I assay [20]. Briefly, four 12-well tissue-culture-treated plates (VWR International, Mississauga, ON, Canada) were seeded with Sf9-Cas9 cells at a density of 0.9 × 10^6^ cells/well and allowed to adhere for ~1 h at 27 °C. The cells were then transfected with either the scrambled control plasmid or *gp64* targeting sgRNA plasmids (*n* = 6) using FuGENE HD (Promega, Madison, WI, USA) transfection reagent according to the manufacturer’s protocol. An additional three wells seeded with the Sf9-Cas9 cells were left untransfected. At 16–24 h post-transfection (hpt), the media from each well was aspirated, and fresh Sf-900^TM^ III SFM containing the p6.9GFP rBEV was added to all the wells to achieve an infection at an MOI of 3 pfu/cell. This resulted in six replicates of the transfected+infected samples for each *gp64* targeting sgRNA plasmid, six of the scrambled control, and three of the infected-only control. Approximately 48 h post-infection (hpi), the cells were harvested by centrifugation at 800× *g* for 15 min. Cell cultures from three wells (replicates) for each control and targeted sample were pooled for sequencing. The pooled cell pellets and supernatants were stored at −80 °C and 4 °C, respectively, to confirm mutations in both intracellular and extracellular p6.9GFP rBEV genomes via next-generation sequencing (NGS). Finally, the supernatants from the other replicates (*n* = 3) of the scrambled control and targeted samples were analyzed by EPDA and flow cytometry for infectious and total baculovirus titers, respectively, and the cell pellets via flow cytometry for GFP production.

### 4.5. Flow Cytometry Analysis of GFP upon gp64 Gene Disruption

The cell pellets from the T-I assay were prepared for flow cytometry by resuspending them in 2% paraformaldehyde (Sigma-Aldrich, Oakville, ON, Canada) in phosphate-buffered saline (PBS) (Wisent Inc., Saint-Jean-Baptiste, QC, Canada), followed by incubation at 4 °C for ~30 min. Fixed samples were diluted in 1× PBS before being analyzed using a BD Accuri^TM^ C6 Plus flow cytometer (BD Biosciences, San Jose, CA, USA) equipped with a blue laser with an excitation frequency of 488 nm. Samples were run at a low flow rate of 14 µL/min, with 10,000 events collected per sample. Licensed software, FlowJo^TM^ V10 (Tree Star, Ashland, OR, USA), was used to analyze the acquired flow data. After applying a gate to remove cell debris, a histogram of the gated population was used to bin the fluorescence detected by the FL1-H detector into high (FL1-H ≥ 10^6^ au) and low (FL1-H < 10^6^ au) fluorescence bins, and these gates were applied to all the samples. The R programming language was used to process the data and visualize the results further.

### 4.6. Total Baculovirus Quantification via Flow Cytometry

Total baculovirus particles were quantified by a previously described SYBR Green staining-based flow cytometry assay [46]. Briefly, 10^−2^ diluted virus samples in 1× PBS were fixed in 2% paraformaldehyde at 4 °C for 1 h. This was followed by a freeze-thaw cycle during which samples were subjected to freezing at −80 °C for 30 min and thawing at 27 °C for 10 min. The samples were then treated with 10% Triton X-100 (Sigma-Aldrich, Oakville, ON, Canada) for 5 min to permeabilize the membrane. SYBR Green I Nucleic Acid Gel Stain 10,000× (Thermo Fisher, Mississauga, ON, Canada) diluted to 5 × 10^−3^ was added to the samples to stain dsDNA. The stained samples were incubated in the dark at 80 °C for 10 min, followed by a cooling step on ice for 10 min before being analyzed by flow cytometry.

The prepared samples were analyzed by a BD Accuri^TM^ C6 Plus flow cytometer (BD Biosciences, San Jose, CA, USA) equipped with a blue laser with an excitation frequency of 488 nm. Each sample was run at a medium flow rate of 35 µL/min for 30 s. A detailed flow cytometry analysis protocol for total baculovirus quantification can be found elsewhere [35]. Briefly, acquired data were processed using the FlowJo^TM^ V10 software (Tree Star, Ashland, OR, USA) and the R programming language. For calibration purposes, 3 µm FlowSet fluorospheres (Beckman Coulter, Mississauga, ON, Canada) with a concentration of 1 × 10^6^ fluorospheres/mL were used.

### 4.7. Tiled-Amplicon Sequencing Assay for rBEV Genomes

The sequencing assay was adapted from a previous study [47]. All custom index primers (Supplementary Table S1) and tiled-amplicon primers [21] used in this study were synthesized by IDT. The adaptation of this tiled-amplicon sequencing assay to targeted rBEV genomes has been described elsewhere [21]. Briefly, using PrimalScheme [47], tiled-amplicon primers were designed to amplify the rBEV genomes recovered from the T-I assay. These gDNAs were extracted using the Wizard Genomic DNA Purification kit (Promega, Madison, WI, USA) following the manufacturer’s protocol. Odd-numbered and even-numbered primer pairs were pooled into Primer Pools 1 and 2, respectively, to generate alternate amplicons via multiplex PCR. The size of the amplicons was confirmed by running the PCR products on 1% agarose gel. Subsequently, amplicons from each PCR for a sample were pooled and purified using AMPure XP magnetic beads (Beckman Coulter, Mississauga, ON, Canada) according to the manufacturer’s directions. Purified amplicons were quantified using the Qubit dsDNA high-sensitivity assay kit (Thermo Fisher Scientific, Waltham, MA, USA) following the manufacturer’s instructions, and then prepared for DNA library construction using the Illumina DNA Prep, (M) Tagmentation kit (Illumina, San Diego, CA, USA) according to the manufacturer’s protocol. The indexed amplicons were then quantified using the Qubit dsDNA high-sensitivity assay kit (Thermo Fisher Scientific, Waltham, MA, USA) as described by the manufacturer and visualized on 1% agarose gel to estimate the median sizes of the DNA libraries. Finally, the DNA and PhiX control libraries were denatured and diluted as previously described [21] and loaded onto the MiSeq reagent kit v3 (Illumina, San Diego, CA, USA) cartridge for sequencing.

### 4.8. Bioinformatics Pipeline for Minor Species

The variant calling pipeline (included in the Supplementary), with a reference genome and two technical replicates for each sample, was run from the Ubuntu terminal command prompt (22.04.5 LTS). Briefly, for running the pipeline with a reference, the ‘bwa index reference.fasta’ [48] command was used to prepare the FASTA file for alignment by creating index files of the reference genome with extensions .amb, .ann, .bwt, .pac, and .sa. Following this, *bwa mem* [48] was utilized to map a sample’s forward and reverse sequencing reads to the indexed reference, creating a sam file. Using *samtools view* [34], with –q and –F parameter thresholds 10 and 2308, respectively, the sam file was converted to a raw bam file. This raw bam file was then realigned to the reference genome by *lofreq viterbi* [49] to correct mapping errors, especially around indels. The realigned bam file was sorted by *samtools sort* [34] based on its position in the reference genome determined through alignment. For fast random access of data, the sorted bam file was index coordinated using *samtools index* [34]. *Samtools ampliconclip* [34] was then utilized to clip primers from reads in the sorted bam file with the –filter-len parameter set to 200, and the output was a trimmed bam file. Subsequently, *samtools mpileup* [34] generated a pileup file from the trimmed bam file, which summarized the alignment of reads to the reference genome. The *samtools mpileup* output was piped into *iVar variants* [33] to detect variants (single-nucleotide variants and indels) with the –q parameter set to 20 and –t set to 0.01. To correctly call variants, the reference genome was passed using the –r flag, and after processing the pileup file, a tab-delimited file was generated with variant details. Finally, *iVar filtervariants* [33] was used to filter variants across the .tsv files of the technical replicates of a sample to create a single filtered variant file for the sample (Supplementary Table S2 describes the .tsv file column headers). All the .tsv files were then analyzed using the R programming language.

## 5. Conclusions

All targeted disruption of the AcMNPV *gp64* gene at different locations reduced BV co-production in cell culture while enhancing GFP production. Our results suggest improved disruption when multiple targeting sites are used. Our sequencing pipeline successfully determined the resulting CRISPR-Cas9-mediated mutations within *gp64* for the majority of the targets (gp64+131, gp64−160, gp64+378, and gp64+418). Additionally, our analysis suggested that there were no off-target effects and confirmed that variants could be conserved over virus amplification in cell culture.

## Figures and Tables

**Table 1:** Overview of true deletion mutations (Fisher’s exact test *p*-value ≤ 0.05) and corresponding variant frequencies at different locations within the *gp64* gene of the p6.9GFP rBEV genomes recovered from the CRISPR-Cas9-based transfection-infection assay (T-I assay).

Target ^1^	Estimated Position (bp) ^2^	Observed Position (bp) [Mutation] (Frequency) ^3^	
		Cell Pellet	Supernatant
gp64+131	107,222–107,227	107,222 [−C] (6.62%)	Not observed
gp64−160	107,199–107,204	107,201 [−CCA] (6.30%) 107,202 [−CA] (8.47%)	107,199 [−CTCCA] (3.05%) 107,200 [−TCCA] (3.48%) 107,201 [−CCA] (5.44%) 107,202 [−CA] (6.34%) 107,204 [−C] (1.43%)
gp64+278	107,075–107,080	Not observed	Not observed
gp64+378	106,975–106,980	106,975 [−T] (4.51%)	106,975 [−T] (4.04%)
gp64+418	106,935–106,940	106,935 [−CA] (4.24%)	106,935 [−CA] (2.17%)
gp64+131/384	107,222–107,227 106,969–106,974	Not observed	Not observed

^1^ sgRNA targeting *gp64* at a specific location within the *gp64* gene; ^2^ position of 1–5 bp indel mutations in *gp64* that could occur 3–4 bp upstream of the protospacer adjacent motif (PAM) upon CRISPR-Cas9 targeting; ^3^ position of true mutations within *gp64*, true deletion mutations within *gp64*, and frequencies at which those true mutations occur in both cell pellet and supernatant fractions, as obtained from tiled-amplicon sequencing.

**Table 2:** Conserved variants found in different samples outside the homologous repeat regions (hrs).

Mutation Region	Mutation Type	Virus Stock Consensus/ Virus Stock Variant/ T-I Assay Variant ^1^	Mutation Position
AcOrf-84 promoter	SNPs	G/T/T	71,443
fgf 3^′^ UTR	SNPs	A/G/G	27,502
fgf 3^′^ UTR	SNPs	G/A/A	27,505
fgf 3^′^ UTR	SNPs	G/A/A	27,506
fgf 3^′^ UTR	SNPs	G/A/A	27,509
*AcOrf-603*	SNPs	C/T/T	3960
*AcOrf-1629*	SNPs	G/A/A	6375
*lef10*	SNPs	G/A/A	45,761
*AcOrf-91*	SNPs	T/A/A	78,627
*AcOrf-91*	SNPs	A/T/T	78,666
*AcOrf-1629*	Insertion	-/GATC/GATC	7304
*AcOrf-51*	Insertion	-/A/A	44,073
*egt*	Deletion	^2^ CTAGAGA/-/-	12,427
*AcOrf-91*	Deletion	TAT/-/-	78,926

^1^ Changes in nucleotides between the reference genome on the left, the variants in the shotgun sequenced virus stock in the middle, and the variants in the tiled-amplicon sequenced rBEV genomes recovered from the T-I assay on the right; ^2^ CTAGAGATCTCTAGAGATCT, the complete 20 bp deletion sequence observed in the *egt* gene.

**Table 3:** Spacer sequences used for control and AcMNPV *gp64* gene.

Gene	Location	Spacer Sequence (5^′^-3^′^)	PAM	Strand
Scrambled control	N/A	CACCTTGAAGCGCATGAACT	N/A	N/A
*gp64*	+131	GGAAACGCTGCAAAAGGACG	TGG	Antisense
*gp64*	−160	GTTGTAGTCCGTCTCCACGA	TGG	Sense
*gp64*	+278	AACGCTGAATGTGGGCAAAG	AGG	Antisense
*gp64*	+378	GACTGTTTTCGCGACAACGA	GGG	Antisense
*gp64*	+418	AAGGCAAAGAGTTGGTGAAG	CGG	Antisense
*gp64*	+131/ 384	GGAAACGCTGCAAAAGGACG/ TTTCGCGACAACGAGGGCCG	TGG/ CGG	Antisense

## Data Availability

The datasets generated during and/or analyzed during the current study are available from the corresponding author on reasonable request. All the tab-delimited files generated utilizing the variant calling pipeline have been submitted with Borealis, The Canadian Dataverse Repository (https://doi.org/10.5683/SP3/YEECMU) [50] (accessed on 8 May 2025).

## Supplementary Materials

The following supporting information can be downloaded at https://www.mdpi.com/article/10.3390/ijms26125805/s1.

## Abbreviations

The following abbreviations are used in this manuscript:

AcMNPV	*Autographa californica* multiple nucleopolyhedrovirus
au	arbitrary units
BEVS	baculovirus expression vector system
DIPs	defective interfering particles
EPDA	end-point dilution assay
GFP	green fluorescent protein
HDR	homology-directed repair
hpi	hours post-infection
hpt	hours post-transfection
hrs	homologous repeat regions
IDT	integrated DNA technologies
IVT	infectious virus titer
MOI	multiplicity of infection
NHEJ	non-homologous end joining
NGS	next-generation sequencing
ORF	open reading frame
ori	origin of replication
PAM	protospacer adjacent motif
PBS	phosphate-buffered saline
pfu/mL	plaque-forming units per mL
rBEV	recombinant baculovirus expression vector
SFM	serum-free media
sgRNA	single-guide RNA
T-I assay	transfection-infection assay
