# Supplementary material for: Baculovirus Variant Detection from Transient CRISPR-Cas9-Mediated Disruption of gp64 at Different Gene Locations
Source: Int J Mol Sci. 2025 Jun 17;26(12):5805. doi: 10.3390/ijms26125805 (PMC12193402; doi:10.3390/ijms26125805)
Supplement: Supplementary file 1 [file ijms-26-05805-s001.zip › ijms-3656758-supplementary.pdf]

Supplementary Materials: Baculovirus Variant Detection from Transient CRISPR-Cas9-Mediated Disruption of *gp64* at Different Gene Locations

Madhuja Chakraborty [1], Lisa Nielsen [1,2], Delaney Nash [2], Mark R. Bruder [1], Jozef I. Nissimov [2], Trevor C. Charles [2] and Marc G. Aucoin [1,*]

Table S1. Custom primers (unique indexes with sequences appended to their 5′ and 3′ ends) used in this study to construct MiSeq DNA libraries. These unique i7 and i5 index pairs for each sample are read by the machine to distinguish reads from different samples.

Target	Replicate	i7 Primer ID	i7 Index (5′-3′)†	i5 Primer ID	i5 Index (5′-3′)‡
gp64+131_C	A	N721	GCAGCGTA	S515	TTCTAGCT
gp64+131_C	B	N722	CTGCGCAT	S515	TTCTAGCT
gp64+131_S	A	N721	GCAGCGTA	S516	CCTAGAGT
gp64+131_S	B	N722	CTGCGCAT	S516	CCTAGAGT
gp64-160_C	A	N723	GAGCGCTA	S515	TTCTAGCT
gp64-160_C	B	N724	CGCTCAGT	S515	TTCTAGCT
gp64-160_S	A	N723	GAGCGCTA	S516	CCTAGAGT
gp64-160_S	B	N724	CGCTCAGT	S516	CCTAGAGT
gp64+278_C	A	N714	TCATGAGC	S515	TTCTAGCT
gp64+278_C	B	N715	CCTGAGAT	S515	TTCTAGCT
gp64+278_S	A	N714	TCATGAGC	S516	CCTAGAGT
gp64+278_S	B	N715	CCTGAGAT	S516	CCTAGAGT
gp64+378_C	A	N716	TAGCGAGT	S515	TTCTAGCT
gp64+378_C	B	N718	GTAGCTCC	S515	TTCTAGCT
gp64+378_S	A	N716	TAGCGAGT	S516	CCTAGAGT
gp64+378_S	B	N718	GTAGCTCC	S516	CCTAGAGT
gp64+418_C	A	N719	TACTACGC	S515	TTCTAGCT
gp64+418_C	B	N720	AGGCTCCG	S515	TTCTAGCT
gp64+418_S	A	N719	TACTACGC	S516	CCTAGAGT

Table S1 continued from previous page

Target	Replicate	i7 Primer ID	i7 Index (5′-3′)†	i5 Primer ID	i5 Index (5′-3′)‡
gp64+418_S	B	N720	AGGCTCCG	S516	CCTAGAGT
gp64+131/384_C	A	N727	ACTGATCG	S515	TTCTAGCT
gp64+131/384_C	B	N729	GACGTCGA	S515	TTCTAGCT
gp64+131/384_S	A	N727	ACTGATCG	S516	CCTAGAGT
gp64+131/384_S	B	N729	GACGTCGA	S516	CCTAGAGT
ScrambledControl_C	A	N716	TAGCGAGT	S517	GCGTAAGA
ScrambledControl_C	B	N718	GTAGCTCC	S517	GCGTAAGA
ScrambledControl_S	A	N714	TCATGAGC	S517	GCGTAAGA
ScrambledControl_S	B	N715	CCTGAGAT	S517	GCGTAAGA
Infected-onlyControl_C	A	N721	GCAGCGTA	S517	GCGTAAGA
Infected-onlyControl_C	B	N722	CTGCGCAT	S517	GCGTAAGA
Infected-onlyControl_S	A	N719	TACTACGC	S517	GCGTAAGA
Infected-onlyControl_S	B	N720	AGGCTCCG	S517	GCGTAAGA

† All i7 indexes have the sequence CAAGCAGAAGACGGCATACGAGAT appended to their 5′ end and GTCTCGTGGGCTCGG to their 3′ end;
‡ All i5 indexes have the sequence AATGATACGGCGACCACCGAGATCTACAC appended to their 5′ end and TCGTCGGCAGCGTC to their 3′ end.

Variant calling pipeline with a reference genome and two technical replicates:

```
bwa index reference.fasta

for sample in $(cat samples)
do
echo "On Sample: $sample"

bwa mem -t 20 reference.fasta $sample_a_R1.fq.gz $sample_a_R2.fq.gz >
$sample_A.sam

samtools view -b -S -q 10 -F 2308 -o $sample_A-RAW.bam $sample_A.sam

./pkgs/lofreq_star-2.1.2/bin/lofreq viterbi -f reference.fasta
-o $sample_A_realigned.bam $sample_A-RAW.bam

samtools sort -o $sample_A.sort.bam $sample_A_realigned.bam &&
samtools index $sample_A.sort.bam

samtools ampliconclip –both-ends –filter-len 200 -b scheme.primer.bed
$sample_A.sort.bam -o $sample_A.trim.bam

rm $sample_A.sam $sample_A-RAW.bam $sample_A_realigned.bam
$sample_A.sort.bam $sample_A.sort.bam.bai

samtools mpileup -aa -A -d 0 -B -Q 0 -f reference.fasta $sample_A.trim.bam |
ivar variants -p $sample_A_variants -q 20 -t 0.01 -m 0 -r reference.fasta

bwa mem -t 20 reference.fasta $sample_b_R1.fq.gz $sample_b_R2.fq.gz >
$sample_B.sam

samtools view -b -S -q 10 -F 2308 -o $sample_B-RAW.bam $sample_B.sam

./pkgs/lofreq_star-2.1.2/bin/lofreq viterbi -f reference.fasta
-o $sample_B_realigned.bam $sample_B-RAW.bam

samtools sort -o $sample_B.sort.bam $sample_B_realigned.bam &&
samtools index $sample_B.sort.bam

samtools ampliconclip –both-ends –filter-len 200 -b scheme.primer.bed
$sample_B.sort.bam -o $sample_B.trim.bam

rm $sample_B.sam $sample_B-RAW.bam $sample_B_realigned.bam
$sample_B.sort.bam $sample_B.sort.bam.bai

samtools mpileup -aa -A -d 0 -B -Q 0 -f reference.fasta $sample_B.trim.bam |
ivar variants -p $sample_B_variants -q 20 -t 0.01 -m 0 -r reference.fasta

ivar filtervariants -p $sample_variants $sample_A_variants.tsv $sample_B_variants.tsv
done
```

Table S2. Description of the column headers appearing in each .tsv output file obtained from running the variant calling pipeline with a reference genome and two technical replicates. Here, 1_A_variants.tsv and 1_B_variants.tsv represent the .tsv file names for sample 1 with replicates A and B.

Column header	Description
REGION	Common region across all replicate variant .tsv output files
POS	Common position across all variant .tsv output files
REF	Common reference nucleotide across all variant .tsv output files
ALT	Common alternate nucleotide across all variant .tsv output files
GFF_FEATURE	A feature used for translation
REF_CODON	Codon using the reference nucleotide
REF_AA	Translated amino acid from reference codon
ALT_CODON	Codon using the alternate nucleotide
ALT_AA	Translated amino acid from alternate codon
REF_DP_1_A_variants.tsv	Reference nucleotide depth in replicate A
REF_RV_1_A_variants.tsv	Reverse reads reference nucleotide depth in replicate A
REF_QUAL_1_A_variants.tsv	Average quality of reference nucleotide in replicate A
ALT_DP_1_A_variants.tsv	Alternate nucleotide depth in replicate A
ALT_RV_1_A_variants.tsv	Reverse reads alternate nucleotide depth in replicate A
ALT_QUAL_1_A_variants.tsv	Average quality of alternate nucleotide in replicate A
ALT_FREQ_1_A_variants.tsv	Frequency of alternate nucleotide in replicate A
TOTAL_DP_1_A_variants.tsv	Total depth at the position in replicate A
PVAL_1_A_variants.tsv	*p*-value of fisher's exact test in replicate A
PASS_1_A_variants.tsv	*p*-value (≤ 0.05) result in replicate A

Table S2 continued from previous page

Column header	Description
REF_DP_1_B_variants.tsv	Reference nucleotide depth in replicate B
REF_RV_1_B_variants.tsv	Reverse reads reference nucleotide depth in replicate B
REF_QUAL_1_B_variants.tsv	Average quality of reference nucleotide in replicate B
ALT_DP_1_B_variants.tsv	Alternate nucleotide depth in replicate B
ALT_RV_1_B_variants.tsv	Reverse reads alternate nucleotide depth in replicate B
ALT_QUAL_1_B_variants.tsv	Average quality of alternate nucleotide in replicate B
ALT_FREQ_1_B_variants.tsv	Frequency of alternate nucleotide in replicate B
TOTAL_DP_1_B_variants.tsv	Total depth at the position in replicate B
PVAL_1_B_variants.tsv	p-value of fisher's exact test in replicate B
PASS_1_B_variants.tsv	p-value (≤ 0.05) result in replicate B

Disclaimer/Publisher's Note: The statements, opinions and data contained in all publications are solely those of the individual author(s) and contributor(s) and not of MDPI and/or the editor(s). MDPI and/or the editor(s) disclaim responsibility for any injury to people or property resulting from any ideas, methods, instructions or products referred to in the content.
